# First Insights Into the LC‐HRMS Profiling and Biological Activities of *Crocus graveolens*

**DOI:** 10.1002/fsn3.70751

**Published:** 2025-08-13

**Authors:** Ezgi Ersoy, Mehmet Boğa, Alevcan Kaplan, Emel Mataracı Kara, Serpil Demirci Kayıran, Esra Eroğlu Özkan

**Affiliations:** ^1^ Department of Pharmacognosy Faculty of Pharmacy, Biruni University Istanbul Türkiye; ^2^ Department of Analytical Chemistry Faculty of Pharmacy, Dicle University Diyarbakır Türkiye; ^3^ Department of Crop and Animal Production Sason Vocational School, Batman University Batman Türkiye; ^4^ Department of Pharmaceutical Microbiology Faculty of Pharmacy, Istanbul University Istanbul Türkiye; ^5^ Cukurova University Faculty of Pharmacy Pharmaceutical Botany Department Adana Türkiye; ^6^ Department of Pharmacognosy, Faculty of Pharmacy Istanbul University Istanbul Türkiye

**Keywords:** biological properties, *crocus graveolens*, LC‐HRMS, saffron

## Abstract

There are documented reports of 103 different *Crocus* species, commonly known as saffron, distributed across Türkiye. Many of these species have been used not only as spices but also in the treatment or prevention of various diseases. However, the majority of research has focused only on *Crocus sativus* L., and only a few other members of the genus have been investigated for their chemical constituents and biological properties. *Crocus graveolens* Boiss. & Reut. is one of those species used against gynecological diseases through oral administration. This is the first report regarding phytochemicals by LC‐HRMS analysis and in vitro biological activities of the species. Accordingly, a total of 33 different secondary metabolites were detected in varying quantities. The major compound was determined as rutin (162.808 ± 4.5 μg/g extract). Total phenolic content (32.79 ± 1.30 μg PEs/mg extract) and total flavonoid content (40.29 ± 0.24 μg QEs/mg) were calculated. The antioxidant activity of the extract was evaluated by three different assays, namely, DPPH free and ABTS cation radical scavenging and CUPRAC activity methods, and moderate activity was demonstrated compared to the standards. The extract inhibited acetylcholinesterase with 30.88% ± 1.51% and butyrylcholinesterase with 61.22% ± 0.47% at a 200 μg/mL concentration. Besides, the extract was effective against *Staphylococcus aureus* , *Staphylococcus epidermidis* , and *Candida albicans* strains. This study features a new contribution to *Crocus* research in Türkiye.

## Graphical abstract

This study reports the first comprehensive analysis of Crocus graveolens Boiss. & Reut. using LC‐HRMS, identifying 33 secondary metabolites, with rutin as the major compound (162.808 ± 4.5 μg/g extract). The extract showed moderate antioxidant activity and effectively inhibited acetylcholinesterase (30.88% ± 1.51%) and butyrylcholinesterase (61.22% ± 0.47%) activities, while also exhibiting antimicrobial effects against *Staphylococcus aureus* , *Staphylococcus epidermidis* , and *Candida albicans* . This research represents a new contribution to Crocus studies in Turkiye.

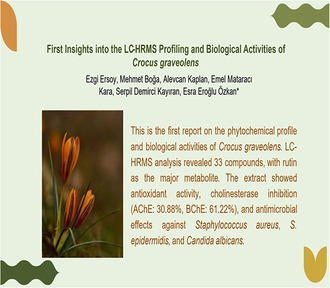

## 1 Introduction

Saffron, the dried red stigmas of *Crocus sativus* L. (Iridaceae), is renowned for having the highest price‐to‐quantity ratio among all spices (Wenger 2022). Given that the plant can only be harvested over a brief period of 15–20 days, the cultivation and processing of saffron necessitate meticulous and manual techniques, and almost 200,000 flowers are needed to produce 1 kg of saffron; this outcome is understandable (Dai et al. 2021). Archaeological studies indicate that these species have been one of the most popular plants since prehistoric times, especially with the finding of 50,000‐year‐old cave art created with pigments derived from saffron. Not only that, but also its use for medicinal purposes as a sense‐forcing, resolving, modifier, diuretic, astringent, tonic, and convoying agent was well‐documented (Yousefi and Shafaghi 2020, Mohtashami et al. 2021). Saffron has a profound significance in Türkiye as well. It has applications in the Turkish traditional medicine system (against gastrointestinal and respiratory diseases, heart‐related complaints, pains, depression, and as a sexual potency enhancer), Turkish cuisine, dyeing, cosmetic products, and even in the literature. There have been many poems, folk riddles, and rhymes about saffron in the Turkish language (Yildirim et al. 2020).

Türkiye boasts a remarkable diversity of *Crocus* species. Among 235 species, 103 of them are distributed here, reflecting its unique geographical and climatic conditions (Yazici et al. 2024). Besides, many of these have been traditionally used for therapeutic purposes. For instance, bulbs, leaves, and aerial parts of *Calochortus leichtlinii* (D. Dewar) Bowles are used against stomach aches and shortness of breath (Kılıç et al. 2020). *C. ancyrensis* (Herbert) Maw flowers are used as diuretics (Günbatan et al. 2016 *). C. danfordiae* Maw corms and *C. kotschyanus* K. Koch aerial parts are applied to wounds to accelerate the healing process (Sargin 2015; Sargin and Büyükcengiz 2019). *Crocus* *graveolens* Boiss. & Reut. is also among the species with ethnopharmacological significance. It is called “çördük” and is used orally against gynecological cysts by local people in the district of Antakya (Güzel et al. 2015).

The research into the chemical and biological properties of *Crocus* species has been focused on *Crocus. sativus* for the most part. A substantial body of research exists about every part of the plant, including extracts prepared with different solvents, phytochemical analyses, in vitro and in vivo pharmacological activities, and even clinical trials. The results of these countless studies rationalized their ethnobotanical use in many aspects. In a nutshell, *C. sativus* extracts were shown to demonstrate anticancer, antidepressant, antidiabetic, antimicrobial, anti‐inflammatory, antioxidant, anticonvulsant, and hypolipidemic activities attributed to their rich phytochemical compositions, including their major carotenoids and crocin derivatives (Bukhari et al. 2018). Over and above, *C. sativus* is considered not only effective but also quite a safe plant in terms of risk–benefit ratio. No adverse effects have been observed at a daily dose of 30 mg/day; even high doses (above 1.5 g/day) did not cause any toxic reactions (Mykhailenko et al. 2019).

As aforementioned, there is a notable scarcity of research on *Crocus* species other than *C. sativus*. Only a few papers have been published regarding the pharmacognostical properties of different *Crocus* species, although they have also been used in traditional medicine by many cultures. *C. graveolens* is among those insufficiently researched species, as pointed out by Mykhailenko et al. (2019). In that comprehensive review, the researchers reported that only the presence of tricin was determined in the leaves of the plant by Bate‐Smith (1968) and Harborne and Williams (1984). This condition represents a significant threat to public health, considering this plant has been used orally by local people of Türkiye. Furthermore, the ethnobotanical significance of *C. graveolens* accentuates the necessity for in‐depth research into its medicinal potential.

With the preceding considerations in mind, this study aimed to provide data about the phytochemical composition and important biological activities of *C. graveolens* . The chemical constituents were analyzed by LC‐HRMS using 65 standards. The antioxidant potential of the plant was evaluated using three different assays. Considering the frequent use of *C. sativus* against cognitive disorders, the anticholinesterase activity of *C. graveolens* was also tested. Additionally, the antimicrobial activity of the plant was investigated against ten pathological strains, including eight bacteria and two fungi. The authors hope to enrich the existing limited body of knowledge, offering new insights that could pave the way for future research and practical applications about this valuable plant.

## 2 Materials and Methods

### 2.1 Plant Material and Extraction

The whole plant of *Crocus graveolens* Boiss. & Reut. was collected from Alanlı Village, Andırın–Kahramanmaraş/Türkiye at an elevation of 800 m in April 2023. The identification of the species was validated by Assoc. Prof. Dr. Serpil Demirci Kayiran from the Pharmaceutical Botany Department at Çukurova University's Faculty of Pharmacy. A herbarium voucher specimen has been deposited at the Çukurova University Faculty of Pharmacy Herbarium, cataloged under CUEF number 766. For the extraction process, 10 g of whole plant was macerated in 100 mL of ethanol for 24 h at room temperature. Following this, the mixture was filtered using Whatman No. 1 filter paper. This extraction procedure was carried out three times to maximize yield. The combined filtrates were concentrated under reduced pressure using a rotary evaporator at 40°C. The resulting concentrated extract was stored at −20°C until needed for bioactivity assays and LC‐HRMS analysis.

### 2.2 LC‐HRMS Analysis

The LC‐HRMS analyses were performed on a Thermo Orbitrap Q‐Exactive mass spectrometer (Thermo Fisher Scientific, Waltham, MA, USA) with a Troyasil C18 column (150 × 3 mm i.d., 5 μm particle size). The method, adapted from Sarikahya Boke et al. (2024), allowed for the identification of compounds by comparing their retention times with standard compounds of 95%–99% purity. High‐resolution mass spectrometry (HRMS) data from the Bezmialem Vakif University Drug Application and Research Center Library (ILMER) were used as a reference.

### 2.3 Antioxidant Activity

#### 2.3.1 Total Phenolic and Flavonoid Contents of the Extract

The total phenolic content was calculated as microgram of pyrocatechol equivalents (PEs); total flavonoid content was calculated as μg of quercetin equivalents (QEs) by applying the method previously used by Boğa et al. (2021).

The total phenolic content results were computed using the following equation:

$$\text{Absorbance}=0.0272\;\text{pyrocatechol}\;\left(\mathrm{\mu g}\right)+0.04466\;\left(r^{2}=0.9989\right)$$

The total flavonoid content results were computed using the following equation:

$$\text{Absorbance}=0.0278\;\text{quercetin}\;\left(\mathrm{\mu g}\right)+0.039\;\left(r^{2}=0.9952\right)$$

#### 2.3.2 Antioxidant Activity Assays

The antioxidant properties of the *C. graveolens* extract were investigated through several in vitro antioxidant activity assays, namely, DPPH free radical scavenging, ABTS cation radical decolorization, and CUPRAC activity. All details of the procedures were explained in Ersoy et al.'s (2020) study.

### 2.4 Anticholinesterase Activity

The anticholinesterase activity of *C. graveolens* extract was assessed using the method described by Boğa et al. (2021). The absorbance of the reaction mixture was measured at 412 nm. Galantamine, a well‐known cholinesterase inhibitor, served as the positive control to compare activity levels.

### 2.5 Antimicrobial Activity

The antimicrobial activity was tested using the microbroth dilution technique, a widely accepted method for determining antimicrobial susceptibility. Ten pathogenic strains were examined, including *Staphylococcus aureus* ATCC 29213, *S. epidermidis* ATCC 12228, *Enterococcus faecalis* ATCC 29212, *Pseudomonas aeruginosa* ATCC 27853, *Escherichia coli* ATCC 25922, *Klebsiella pneumoniae* ATCC 4352, *Proteus mirabilis* ATCC 14153, *Candida albicans* ATCC 10231, *C. parapsilosis* ATCC 22019, and *Cirrenalia tropicalis* ATCC 750. Clinical and Laboratory Standards Institute protocols were followed (Clinical and Laboratory Standards Institute 1997, 2006, 2010). The minimum inhibitory concentration (MIC) was identified as the lowest concentration that prevented visible growth. The tests were conducted in triplicate, with standard antibacterial and antifungal agents, such as cefuroxime‐sodium, cefuroxime, ceftazidime, amikacin, and clotrimazole, included as controls.

### 2.6 Statistical Analysis

All data on all antioxidant activity and enzyme inhibition activity tests are the average of triplicate analyses. The data were recorded as means ± standard deviations. An analysis of variance was performed by one‐way ANOVA procedures. Significant differences between means were determined by the Student *t*‐test, with *p*‐values of < 0.05 being regarded as significant.

## 3 Results and Discussion

### 3.1 LC‐HRMS Analysis

**FIGURE 1:**
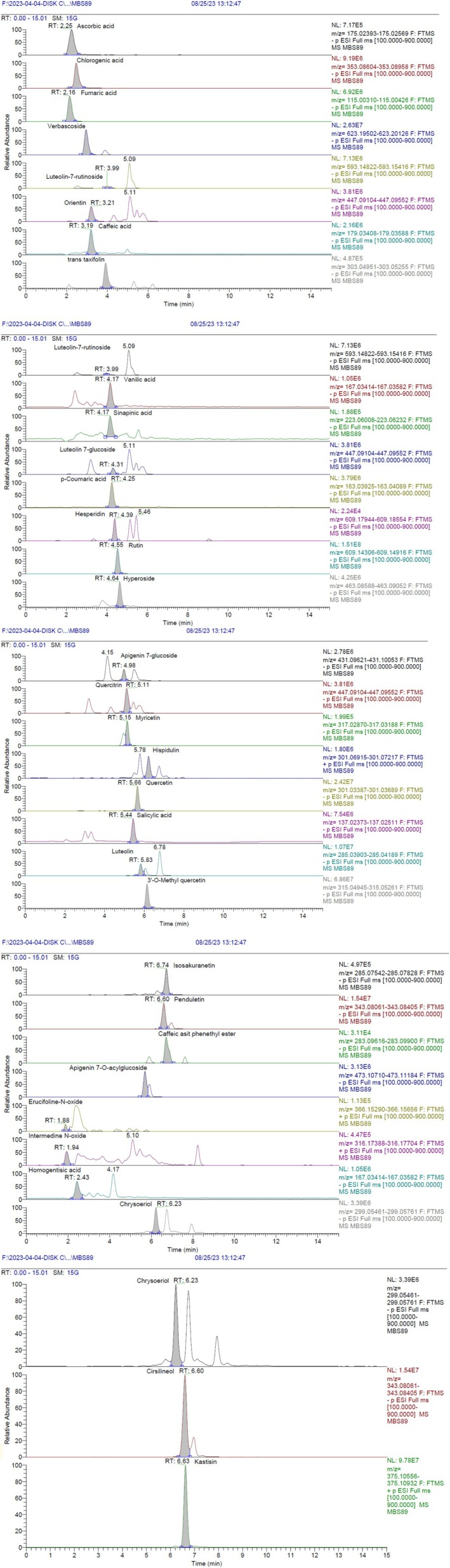
LC‐HRMS chromatograms of the *C. graveolens* extract.

A comprehensive LC‐HRMS analysis was conducted to identify the secondary metabolites of the extract. The chromatograms are displayed in Figure 1, with the corresponding data summarized in Table 1.

**TABLE 1:** LC‐HRMS results of the extract.

No	Compounds	Content of extracts (μg/g)
1	Ascorbic acid	1.447 ± 0.06
2	Chlorogenic acid	5.44 ± 0.19
3	(+)‐Catechin	N.D.
4	Fumaric acid	52.084 ± 1.5
5	(−)‐Epicatechin gallat	N.D.
6	(−)‐Epigallocatechin	N.D.
7	(−)‐Epicatechin gallat	N.D.
8	Verbascoside	14.801 ± 0.43
9	Orientin	1.276 ± 0.05
10	Chicoric acid	N.D.
11	Caffeic acid	0.234 ± 0.00
12	Caffeine	N.D.
13	(+)‐trans taxifolin	0.072 ± 0.00
14	Luteolin‐7‐rutinoside	0.277 ± 0.00
15	Vanillic acid	2.765 ± 0.1
16	Naringin	N.D.
17	Sinapinic acid	15.457 ± 0.55
18	Luteolin‐7‐glycoside	0.236 ± 0.00
19	*p*‐coumaric acid	45.752 ± 1.51
20	Hesperidin	0.013 ± 0.00
21	Rutin	162.808 ± 4.5
22	Syringic acid	N.D.
23	Rosmarinic acid	N.D.
24	Hyperoside	2.165 ± 0.07
25	Dihyrdokaempferol	N.D.
26	Apigenin‐7‐glycoside	0.166 ± 0.00
27	Nepetin‐7‐glycoside	N.D.
28	Ellagic acid	N.D.
29	Quercitrin	0.839 ± 0.03
30	Myricetin	0.032 ± 0.00
31	Quercetin	1.556 ± 0.05
32	Salicylic acid	0.766 ± 0.01
33	Naringenin	N.D.
34	Luteolin	0.138 ± 0.00
35	Genistein	N.D.
36	Nepetin	N.D.
37	3′‐O‐methyl quercetin	2.28 ± 0.08
38	Hispidulin	0.223 ± 0.00
39	Isosakuranetin	0.072 ± 0.00
40	Penduletin	2.153 ± 0.07
41	Caffeic acid phenethyl ester	0.001 ± 0.00
42	Rhamnocitrin	N.D.
43	Chrysin	N.D.
44	Acacetin	N.D.
45	Erucifolin‐N‐oxide	0.304 ± 0.01
46	Europine‐N‐oxide	N.D.
47	Intermedine‐N‐oxide	0.043 ± 0.00
48	Lithospermic acid	N.D.
49	Homogentisic acid	0.489 ± 0.02
50	Cynarin	N.D.
51	Pyrocatechol	N.D.
52	Pyrogallol	N.D.
53	2,5‐dihydro benzoic acid	N.D.
54	Hispidulin‐7‐glycoside	N.D.
55	Dihydrocaffeic acid	N.D.
56	6‐OH‐luteolin‐7‐glycoside	N.D.
57	6‐OMe‐luteolin‐7‐glycoside	N.D.
58	Luteolin‐7‐O‐acyl glycoside	N.D.
59	Apigenin‐7‐O‐acyl glycoside	0.724 ± 0.02
60	Genkwanin	N.D.
61	Chrysoeriol	0.136 ± 0.00
62	Cirsimaritin	N.D.
63	Cirsilineol	1.557 ± 0.06
64	Apigenin‐7‐methylate	N.D.
65	Casticin	1.841 ± 0.12

Abbreviation: N.D., Not detected.

Within the extract, 33 different secondary metabolites were detected in varying quantities. Among them, the major constituent was determined as rutin (162.808 ± 4.5 μg/g extract). Fumaric acid (52.084 ± 1.5 μg/g extract), *p*‐coumaric acid (45.752 ± 1.51 μg/g extract), sinapinic acid (15.457 ± 0.55 μg/g extract), and verbascoside (14.801 ± 0.43 μg/g extract) were also detected in notable quantities in the extract. Moreover, ascorbic acid, chlorogenic acid, orientin, caffeic acid, (+)‐trans taxifolin, luteolin, luteolin‐7‐rutinoside, luteolin‐7‐glycoside, vanillic acid, hesperidin, hyperoside, apigenin‐7‐glycoside, apigenin‐7‐O‐acylglycoside, quercetin, 3′‐O‐methyl quercetin, quercitrin, myricetin, salicylic acid, hispidulin, penduletin, isosakuranetin, erucifolin‐N‐oxide, intermedine‐N‐oxide, homogentisic acid, chrysoeriol, cirsimaritin, and casticin were also identified in the extract.

Prior data on the phytochemical composition of *C. graveolens* are almost nonexistent. To the authors' best knowledge, the only information is provided by Mykhailenko et al. (2019) 's comprehensive review, where they mentioned that tricin was detected in the leaves of the plant.

Nevertheless, many other *Crocus* species were investigated for their secondary metabolites. Accordingly, the chemical compositions of different *Crocus* species were found to be similar, but their concentrations in the extracts differ significantly. A variety of biologically active molecules were shown to be present in different *Crocus* extracts, including carotenoids, monoterpenoids, sesquiterpenoids, flavonoids, anthocyanins, phenylpropanoids, phenolic glycosides, and coumarins. Undoubtedly, the main focus of research has been on the most popular species of the genus, *C. sativus* . Crocin, picrocrocin, and safranal are the three main constituents of *C. sativus* stigma extracts. These compounds were reported to be responsible for the distinguishing color, odor, and bitter taste of saffron (Butnariu et al. 2022). Flavonoids were reported to be more abundant in other organs, such as petals and flowers of the plant. Among them, rutin, kaempferol, and quercetin derivatives, naringenin, rhamnetin, and isorhamnetin were predominantly determined (Termentzi and Kokkalou 2008; Sánchez‐Vioque et al. 2016; Mykhailenko et al. 2022). Regarding other phenolics, chlorogenic acid, *p*‐coumaric acid, vanillic acid, and salicylic acid were detected (Li et al. 2004; Esmaeili et al. 2011; Gismondi et al. 2012).

The data about other *Crocus* species are rather scarce in this context. *C. chrysanthus* Herb. flower methanol extract was characterized mainly by apigenin, isorhamnetin, luteolin, and quercetin (Zengin et al. 2019). In *Crocus. pallasii* subsp. *haussknechtii* (Boiss. & Reut. ex Maw) B. Mathew. extract, apigenin, and kaempferol were screened predominantly (Moudi et al. 2020). In *C. pallasii* Goldb. flower extract, *p*‐coumaric acid, kaempferol derivatives, quercetin, and myricetin were among the detected compounds (Zengin et al. 2020). Siracusa et al. (2022) carried out a detailed analysis of five *Crocus* species collected from different areas in Italy. Reportedly, kaempferol, quercetin, and isorhamnetin were the most abundant components, ranging from 2.7 to 164.7 mg/g. *C. alatavicus* Regel & Semen was found to be rich in phenolics such as gallic acid, chlorogenic acid, vanillic acid, ferulic acid, and *p*‐coumaric acid, kaempferol derivatives, and quercetin (Satybaldiyeva et al. 2016; Allambergenova et al. 2022). An HPLC analysis was conducted on *C. speciosus* Bieb. leaves extract, and chlorogenic acid, mangiferin, isoorientin, hyperoside, isoquercitrin, and kaempferol were identified (Mykhailenko et al. 2021). An HPLC analysis of the methanol extract of *C. baytopiorum* B. Mathew revealed the presence of *p*‐coumaric acid, apigenin‐glucoside, rosmarinic acid, quercetin, and kaempferol (Acar et al. 2010). *C. caspius* was also analyzed by HPLC, and catechin (8.403 mg/g plant) and rutin (1.7 mg/g plant) were determined to be the most abundant (Alizadeh et al. 2023). Different extracts of *C. ancyrensis* Herb. flowers were found to contain rutin as the major compound (Kayir et al. 2023). This is an interesting finding since rutin was revealed as the most abundant compound in the current study, as well. Unquestionably, more studies on the phytochemical profiles of different *Crocus* species are most needed to be able to compare the results with *C. sativus* L.

### 3.2 Total Phenolic and Flavonoid Contents, and Antioxidant Activity

The total phenolic and flavonoid contents of the *C. graveolens* extract were calculated. The antioxidant activity of the extract was assessed using three well‐known methods: DPPH (2,2‐diphenyl‐1‐picrylhydrazyl) radical scavenging, ABTS (2,2′‐azinobis‐(3‐ethylbenzothiazoline‐6‐sulfonic acid)) cation scavenging, and CUPRAC (cupric ion reducing antioxidant capacity). The results can be seen in Table 2.

**TABLE 2:** Total phenolic and flavonoid content, and antioxidant activities of the extract* (Figures 2, 3, 4).

Samples	Total phenolic content (*μ*g PEs/mg extract)^a^	Total flavonoid content (*μ*g QEs/mg extract)^b^	IC_50_ values (μg/mL)^c^		A_0.5_ values (μg/mL)^d^
			DPPH free radical	ABTS cation radical	CUPRAC
*C. graveolens* extract	32.79 ± 1.30	40.29 ± 0.24	74.65 ± 0.70	47.26 ± 0.27	64.61 ± 0.39
BHA^e^	—	—	3.22 ± 0.08	2.74 ± 0.03	4.14 ± 0.17
α‐TOC^e^	—	—	1.41 ± 0.04	8.48 ± 0.43	13.64 ± 0.32
BHT^e^	—	—	16.71 ± 0.80	4.44 ± 0.30	3.93 ± 0.24

* Values expressed are means ± standard deviation of three parallel measurements (*p* < 0.05).

^a^ PEs, pyrocatechol equivalents (*y* = 0.0272*x* + 0.04466 *r* ^2^ = 0.9989).

^b^ QEs, quercetin equivalents (*y* = 0.0278*x* + 0.039 *r* ^2^ = 0.9952).

^c^ Values were given as IC_50_ for DPPH free and ABTS cation radical scavenging activities.

^d^ Values were given as A_0.5_ for CUPRAC activity.

^e^ Standard compounds.

The total phenolic content of the extract is 32.79 ± 1.30 μg PEs/mg extract (*y* = 0.0272*x* + 0.04466 *r* ^2^ = 0.9989), and the total flavonoid content of the extract is 40.29 ± 0.24 μg QEs/mg extract (*y* = 0.0278*x* + 0.039 *r* ^2^ = 0.9952). Interestingly, the total flavonoid content of the extract was found to be higher than the total phenolic content. This may be a result of the high rutin content detected in the extract.

**FIGURE 2:**
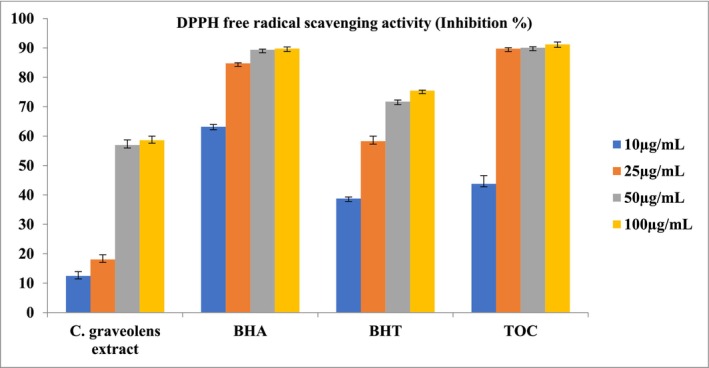
Free radical scavenging activity of the *C. graveolens* extracts, BHA, BHT, and α‐TOC. Values are means ± S.D. of three parallel measurements.

**FIGURE 3:**
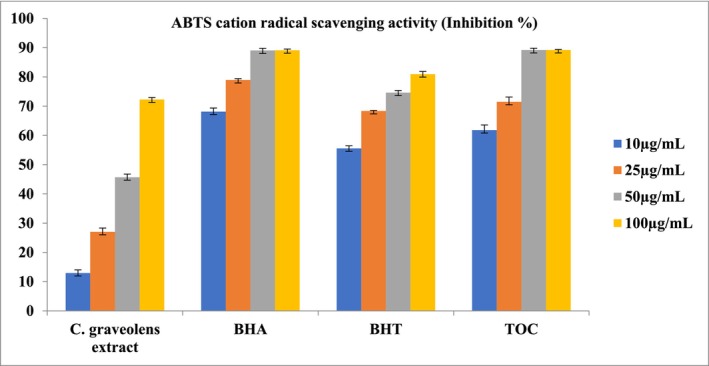
ABTS cation radical scavenging activity of the *C. graveolens* extracts, BHA, BHT, and α‐TOC. Values are means ± S.D. of three parallel measurements.

**FIGURE 4:**
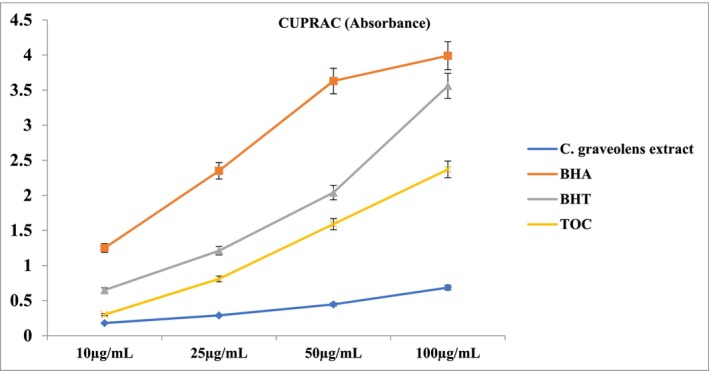
Cupric reducing antioxidant capacity of the *C. graveolens* extracts, BHA, BHT, and α‐TOC. Values are means ± S.D. of three parallel measurements.

Compared to the standards, the extract exhibited moderate antioxidant activity in all three methods. According to the DPPH radical scavenging assay, the IC_50_ value of the extract was calculated as 74.65 ± 0.70 μg/mL (Standards: BHA: 3.22 ± 0.08 μg/mL, α‐TOC: 1.41 ± 0.04 μg/mL, BHT: 16.71 ± 0.80 μg/mL) (Figure 2). The ABTS cation radical scavenging assay was the second procedure, and similarly, the extract demonstrated moderate activity with an IC_50_ value of 47.26 ± 0.27 μg/mL (Standards: BHA: 2.74 ± 0.03 μg/mL, α‐TOC: 8.48 ± 0.43 μg/mL, BHT: 4.44 ± 0.30 μg/mL) (Figure 3). Results of the CUPRAC activity assay also supported this with an A_0.5_ value of 64.61 ± 0.39 μg/mL (Standards: BHA: 4.14 ± 0.17 μg/mL, α‐TOC: 13.64 ± 0.32 μg/mL, BHT: 4.44 ± 0.30 μg/mL) (Figure 4). When evaluating the phytochemical content of *C. graveolens* extract by the LC‐HRMS analysis, alongside the results of total phenolic and flavonoid contents, it can be observed that the extract is not rich in terms of polyphenols. On the other hand, the moderate activity may be attributed to rutin with a lesser contribution from other phenolics.

As expected, the antioxidant properties of *Crocus* species are mostly reported on *C. sativus* L. extracts, and antioxidant research has been focused on the main constituents, namely, crocin, crocetin, and safranal, rather than flavonoids. These three molecules have been shown to demonstrate antioxidant activity with several mechanisms. Furthermore, the extracts were found to exert stronger activity than the isolated molecules, which indicates that the other constituents, including polyphenols, also contribute to this effect for their synergistic behavior (Boskabady and Farkhondeh 2016). There are numerous investigations undertaken on the antioxidant properties of *C. sativus* L., using different techniques, especially on the stigmas of the plant, and the results are generally similar to each other (Papandreou et al. 2006; Hosseinzadeh et al. 2009; Asdaq and Inamdar 2010; Gismondi et al. 2012; Menghini et al. 2018). Cerdá‐Bernad et al. (2022) compared the antioxidant capacity of *C. sativus L. stigma* s from Spain, Iran, and Greece, and reportedly, they all exhibited significant potential for developing new high‐value ingredients owing to their antioxidant properties and bioactive content. Karimi et al. (2010) evaluated the antioxidant potential of *C. sativus* L., focusing on the phenolic profile of the plant. The extract showed lower activity than the standards due to its low amount of total phenolics and flavonoids.

An intriguing approach was brought by Serrano Diaz et al. (2012) in this context. They pointed out that although tepals of the plant had the highest phenolic content, stamens and whole flowers were more effective in terms of antioxidant potential. For that reason, they suggested using not only the stigmas but also other parts of the plant as functional ingredients in the food industry. Baba et al. (2015) also drew attention to this situation when they investigated the antioxidant activity of different parts of *C. sativus* L. The stigmas were the most active part, but also the corms and leaves were found to be sufficiently effective. In another study, the total phenolic and flavonoid contents of *C. sativus* L. petals were higher than those of whole flowers, stamens, and styles, respectively (Jadouali et al. 2019). In Lahmass et al. (2018)'s study, the activity of the stigmas was the lowest, whereas the spaths extract was revealed as the most active.

On the subject of the antioxidant capacity of other *Crocus* species, Khalili et al. (2016) investigated the impact of extraction methods on the antioxidant activity of *C. caspius* Fisch. & C.A. Mey. ex Hohen. Findings suggested that all extracts showed good antioxidant activity with slight differences. *C. mathewii* also exhibited significant antioxidant activity according to β‐carotene–linoleic acid, DPPH, ABTS^+^, CUPRAC, and metal chelating activity assays (Yildiztekin et al. 2016). In another study, Zengin et al. (2020) studied the antioxidant capacity of different parts of *C. pallasii* Goldb., and root extract was found to demonstrate the strongest activity. Zengin et al. (2019) also employed six bioassays to determine the antioxidant properties of different *C. chrysanthus* (Herbert) Herbert extracts. Water extract was revealed as the strongest in terms of antioxidant activity. The investigation of the antioxidant activity of *C. ancyrensis* Maw results also revealed that the water extract displayed the highest activity, which was attributed to rutin and p‐hydroxybenzoic acid found in the extracts (Kayir et al. 2023). On the contrary, Horozić et al. (2020) reported that the ethanol extracts of *C. vernus* (L.) Hill exhibited the highest antioxidant activity, while the aqueous extracts demonstrated the lowest. Moreover, Shakeri et al. (2019) compared the antioxidant capacities of *C. sativus* L. and *C. pallasii* subsp. *haussknechtii* (Boiss. & Reut. ex Maw) B. Mathew, and *C. sativus* L. extracts were shown to demonstrate higher activity.

Taking all into account, it must be noted that the current study was conducted on the extract of *C. graveolens* . Further experiments with analyses on different parts of the plant would be helpful toward a comprehensive understanding of the real potential of these species.

### 3.3 Anticholinesterase Activity

The enzyme inhibitory activity of *C. graveolens* extract was carried out on acetylcholinesterase and butyrylcholinesterase enzymes by applying Ellman et al. (1961)'s method. The results are presented in Table 3.

**TABLE 3:** Acetylcholinesterase and butyrylcholinesterase inhibition (%)^a^ values of the extract.

Samples	Inhibition % (μg/mL)	
	Acetylcholinesterase	Butyrylcholinesterase
*C. graveolens* extract	30.88 ± 1.51	61.22 ± 0.47
Galantamine^b^	91.01 ± 0.22	80.46 ± 0.18

^a^ 200 μg/mL.

^b^ Standard compound.

*C. graveolens* extract demonstrated good butyrylcholinesterase inhibitory (61.22% ± 0.47%) and moderate acetylcholinesterase inhibitory (30.88% ± 1.51%) compared to the standard molecule, galantamine (80.46% ± 0.18% and 91.01% ± 0.22%, respectively) at 200 μg/mL concentration. Ersoy et al. (2020) mentioned that quercitrin, isoquercitrin, hyperoside, and rutin are the major flavonoids responsible for anticholinesterase activity. As aforementioned, *C. graveolens* extract contains a considerable amount of rutin (162.808 ± 4.5 μg/g extract), but only negligible amounts of quercitrin (0.839 ± 0.03 μg/g extract) and hyperoside (2.165 ± 0.07 μg/g extract). Hence, the exhibited cholinesterase inhibitory activity of the extract is possibly due to rutin itself.

*C. sativus* L. is among the medicinal plants traditionally used against dementia (Ayati et al. 2020); its anti‐Alzheimer's potential has been investigated by many researchers. In terms of in vitro and in silico anticholinesterase activity studies, Geromichalos et al. (2012) reported moderate acetylcholinesterase inhibitory activity (up to 30%). According to Menghini et al. (2018)'s study, the *C. sativus* L. extracts prepared with different parts also demonstrated moderate activity. Younis et al. (2023) noted that *C. sativus* L. extract showed significant anticholinesterase activity compared to donepezil, which was attributed to its crocin.

Not only these preliminary studies but also many in vivo and clinical trials have been carried out in this regard. Speaking of which, *C. sativus* L. extracts have been shown to be a candidate to enhance cognitive functions by regulating glutamate levels and tau protein aggregation, also modulating amyloid‐*β* plaques (Farokhnia et al. 2014; Moshiri et al. 2015; Pitsikas 2015; Avgerinos et al. 2020; D'Onofrio et al. 2021; Saeedi and Rashidy‐Pour 2021).

*C. chrysanthus* Herb. extracts were evaluated for their cholinesterase inhibitory activity. Reportedly, ethyl acetate and methanol extracts exerted moderate activity against acetylcholinesterase and butyrylcholinesterase, whereas the water extract was not effective (Zengin et al. 2019). In another study, *C. pallasii* flower, corm, and reticulate fibrous extracts showed moderate cholinesterase inhibitory activity as well (Zengin et al. 2020). On the other hand, none of the extracts prepared with *C. mathewii* Kernd. & Pasche corms and aerial parts demonstrated anticholinesterase activity according to Yildiztekin et al. (2016)'s study.

The inhibition of acetylcholinesterase and butyrylcholinesterase has undeniably been a successful strategy for combating Alzheimer's disease. Many studies indicate the importance of these enzymes as pharmacological targets (Ersoy et al. 2023a, 2023b). Given this, the results of this study may provide new insights into the anti‐Alzheimer potential of *Crocus* species beyond *C. sativus* L., especially considering that these results are consistent with previous studies. Over and above, as *C. sativus* L. has always been promoted as a candidate for the treatment and prevention of dementia, more comprehensive studies should be conducted on other species, including *C. graveolens* .

### 3.4 Antimicrobial Activity

The antimicrobial activity of ethanol extract from *C. graveolens* was evaluated against seven bacterial pathogens and two fungal pathogens for the first time to the authors' best knowledge. The detailed findings, including the minimum inhibitory concentrations (MIC values), are presented in Table 4.

**TABLE 4:** Antimicrobial activity results of the extract.

Microorganisms	MIC Values of the extract (μg/mL)
*P. aeruginosa* ATCC 27853	N.A.
*E. coli* ATCC 25922	N.A.
*K. pneumoniae* ATCC 4352	N.A.
*P. mirabilis* ATCC 14153	N.A.
*S. aureus* ATCC 29213	156.2
*S. epidermidis* ATCC 12228	156.2
*E. faecalis* ATCC 29212	N.A.
*C. albicans* ATCC 10231	312.5
*C. tropicalis* ATCC 750	N.A.
*C. parapsilosis* ATCC 22019	N.A.

*Note:* Standards; Cefuroxime‐Na: 1.2 μg/mL for *S. aureus* ATCC 29213, Cefuroxime 9.8 μg/mL for *S. epidermidis* ATCC 12228, Amikacin 128 μg/mL for *E. faecalis* ATCC29212, Ceftazidime 2.4 μg/mL for *P. aeruginosa* ATCC 27853, Cefuroxime‐Na: 4.9 μg/mL for *E. coli* ATCC 25922 and *K. pneumoniae* ATCC 4352, Cefuroxime‐Na 2.4 μg/mL for *P. mirabilis* ATCC 14153, Clotrimazole 4.9 μg/mL for *C. albicans* ATCC 10231, Clotrimazole 0.25 μg/mL for *C. parapsilosis* ATCC 22019, Clotrimazole 0.25 μg/mL for *C. tropicalis* ATCC 750 Amphotericin B 0.5 μg/mL for *C. parapsilosis* ATCC 22019, Amphotericin B 1 μg/mL for *C. tropicalis* ATCC 750, Amphotericin B 0.5 μg/mL for *C. albicans* ATCC 1023.

Abbreviation: N.A., No Activity.

The results highlighted that *C. graveolens* extract exerted significant antimicrobial activity against *S. aureus* ATCC 29213 and *S. epidermidis* ATCC 12228 with an MIC value of 156.2 μg/mL, and moderate antifungal activity against *C. albicans* ATCC 10231 with 312.5 μg/mL.

In line with expectations, the most investigated member of the *Crocus* genus is *C. sativus* L. also in terms of antimicrobial activity. The tepals of the plant were effective against *Listeria monocytogenes* and *Salmonella* strains even at low concentrations (Kakouri et al. 2017). Using the petals of the plant, hexane, dichloromethane, and ethanol extracts were prepared, and their antimicrobial activity was evaluated against *S. aureus* , *P. aeruginosa* , *E. coli* , and *C. albicans* strains. Among them, the ethanol extract was the strongest one in a dose‐dependent manner (Wali et al. 2020). The most commonly used part, the stigmas of the plant, were also studied to reveal their antimicrobial properties. The results indicated that the water extract exhibited activity against *Acinetobacter baumannii* and *Shigella* sp., achieving an MIC of 600 μg/mL (Drioiche et al. 2023). Lachguer et al. (2023) aimed to enlighten the antimicrobial potential of *C. sativus* L. flower waste to open new possibilities for it to be used in the food and pharmaceutical industries. Evaluating the results, the extracts were notably effective against *S. aureus* strains. Furthermore, the extracts were found to be more effective on Gram‐positive bacteria than on Gram‐negative bacteria.

Research into the antimicrobial activity of other *Crocus* species resulted in several articles. Satybaldiyeva et al. (2015) tested *C. alatavicus* Regel & Semen. against Gram‐positive bacteria. Ethanol extracts from both the aerial parts and corms of *C. alatavicus* demonstrated selective antibacterial activity against *S. aureus, B* *. subtilis* , and *B. cereus* . Significant antimicrobial activities were observed in the methanol and ethyl acetate extracts of *C. biflorus* , *C. baytopiorum*, and *C. flavus* subsp. *dissectus* against *E. coli* , *P. aeruginosa* , *S. aureus* , *Y. enterocolitica* , and *C. albicans* , with MIC values ranging from 0.1 to 25 μg/mL (Acar et al. 2010). *C. caspius* Fisch. & C.A. Mey. ex Hohen. was found to be remarkably effective against all tested strains. The MIC value of 17.08 μg/mL indicated maximum antibacterial activity against *S. aureus* and *A. baumannii* (Alizadeh et al. 2023). Alizadeh et al. (2022) also synthesized gold nanoparticles using *C. caspius* extract, and the nanoparticles exerted a strong antileishmanial activity with an IC_50_ value of 13.92 μg/mL.

Considering all the evidence, phenolic compounds are mainly thought to be responsible for the antimicrobial properties of *Crocus* species. This conclusion was drawn by the high activity of the extracts rich in polyphenols and the comparatively lower activity of the extracts with fewer phenolic compounds. Flavonoids are predominantly the most effective antibacterial agents in this context (Zaazaa et al. 2021; Naim et al. 2022).

## 4 Conclusion

*C. graveolens* can be considered an important plant, especially in two aspects. First, it is closely related to one of the most valuable plant species, *C. sativus* , and second, it has been used traditionally by local people of Türkiye to treat gynecological cysts. However, despite its significance, the scientific data about its efficacy and safety are almost nonexistent. In our current study, it has been found that the extract was rich in rutin, which is a well‐known flavonoid responsible for several health benefits. Owing to rutin and other constituents of the extract, it was observed that the plant has worth mentioning antioxidant, anticholinesterase, and antimicrobial properties. Although this is a preliminary study, it can be considered the first step toward an understanding of the potential of Turkish *Crocus* species other than *C. sativus* .

## Author Contributions

**Ezgi Ersoy:** methodology (equal), project administration (equal), validation (equal), visualization (equal), writing – original draft (equal), writing – review and editing (equal). **Mehmet Boğa:** conceptualization (equal), data curation (equal), formal analysis (equal), investigation (equal), methodology (equal), project administration (equal), supervision (equal), visualization (equal). **Alevcan Kaplan:** investigation (equal), methodology (equal), writing – original draft (equal). **Emel Mataracı Kara:** data curation (equal), investigation (equal), methodology (equal), writing – original draft (equal). **Serpil Demirci Kayıran:** funding acquisition (equal), investigation (equal), methodology (equal), project administration (equal), visualization (equal). **Esra Eroğlu Özkan:** conceptualization (equal), data curation (equal), formal analysis (equal), methodology (equal), project administration (equal), supervision (equal), writing – original draft (equal).

## Conflicts of Interest

The authors declare no conflicts of interest.

## Data Availability

The data that support the findings of this study are available from the corresponding author upon reasonable request.
